# Crosstalk of autophagy and ferroptosis in cardiovascular diseases: from pathophysiology to novel therapy

**DOI:** 10.1016/j.redox.2025.103705

**Published:** 2025-05-29

**Authors:** Changhao Hu, Siying Gao, Xinyi Li, Kaiqing Yang, Ye Cheng, Wei Guo, Huijun Wu, Xueqin Cheng, Weiwen Zhao, Yuxuan Kong, Haoyuan Hu, Songyun Wang

**Affiliations:** Cardiovascular Hospital, Renmin Hospital of Wuhan University, Cardiac Autonomic Nervous System Research Center of Wuhan University, Cardiovascular Research Institute, Wuhan University, Hubei Key Laboratory of Cardiology, China

**Keywords:** Autophagy, Ferroptosis, Cardiovascular diseases, Crosstalk, Therapeutic strategies

## Abstract

Cardiovascular diseases (CVDs) are characterized by high morbidity and mortality rates, imposing substantial epidemiological and economic burdens worldwide. Among the multifaceted mechanisms implicated in CVDs, autophagy and ferroptosis, two intimately linked cellular processes, emerge as pivotal pathophysiological contributors. Autophagy, as an evolutionary conserved process that mediates the degradation and recycling of intracellular components, including proteins and organelles, exerts critical regulatory effects on iron metabolism and lipid homeostasis through various specialized forms, including ferritinophagy and lipophagy. Conversely, ferroptosis, an iron dependent form of cell death, involves oxidative stress and the accumulation of lipid peroxides, often triggered by iron overload and the dysfunction of glutathione peroxidase 4 (GPX4). The intricate crosstalk between these two processes, particularly ferritinophagy-mediated iron regulation influencing ferroptosis, plays a crucial role in diverse CVDs contexts. Key regulatory molecules, such as Beclin-1 and nuclear factor E2-related factor 2 (Nrf2), function as central hubs, orchestrating the intricate interplay between autophagy and ferroptosis. Through a comprehensive examination of these mechanisms across various CVDs pathologies, we summarize the latest findings and outline potential therapeutic strategies targeting the crosstalk between autophagy and ferroptosis. As the inaugural review focusing on autophagy-ferroptosis interactions in CVDs, this work significantly enriches our understanding of the pathophysiology of CVDs and identifies novel therapeutic targets with potential for precision medicine interventions in managing CVDs.

## 1 Introduction

Cardiovascular disease (CVDs) comprises a broad spectrum of pathological alterations, characterized by abnormalities in cardiac structure and function, which has significant morbidity and mortality rates, posing profound health and economic burdens worldwide [1,2]. Although current therapeutic modalities can mitigate the symptoms and progression of CVDs, further investigations are required to elucidate the underlying pathophysiological mechanisms and molecular treatments of CVDs. Increasing experimental evidence indicates that cardiomyocyte homeostatic imbalance and cell death play a significant role in the development and maintenance of CVDs [[3], [4], [5]]. Therefore, it is imperative to establish a comprehensive understanding of the role of physiological homeostasis and cell death to facilitate the effective treatments for CVDs.

As an evolutionarily conserved regulatory mechanism for maintaining cellular homeostasis in eukaryotic cells, autophagy is responsible for segregating, removing and recycling excess proteins and damaged organelles [6,7]. The cellular contents are encapsulated by membrane structures of autophagic vesicles, which subsequently fuse with lysosomes and are eventually broken down into small molecules [8]. In physiological and pathological conditions, autophagy could maintain cellular homeostasis in response to the alterations of microenvironmental triggers, encompassing nutritional, metabolic, physical and chemical factors [9,10]. However, autophagy exerts a double-edged sword effect in CVDs. Appropriate level of autophagy can exert a cytoprotective effect. However, autophagic insufficiency or excessive autophagic flux have been demonstrated to result in autophagy-dependent cell death, thus exacerbating the progression of CVDs [11,12].

**Fig. 1:**
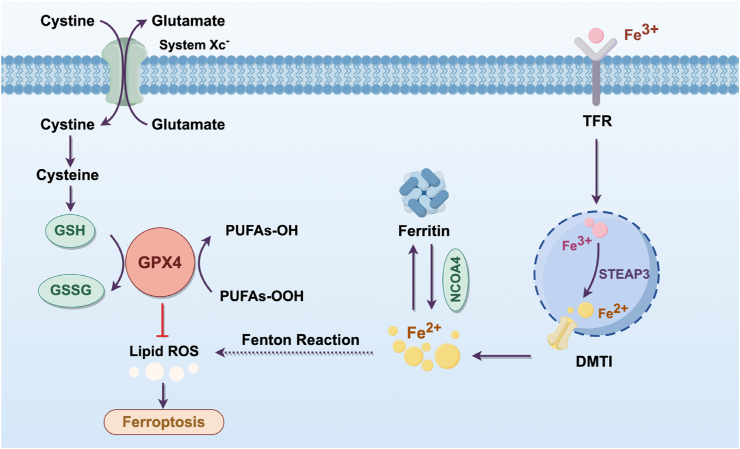
The mechanisms of ferroptosis. Cystine is imported via the System Xc^−^ transporter and reduced to cysteine, fueling glutathione (GSH) synthesis. GPX4, dependent on GSH, neutralizes lipid peroxides (e.g., PUFAs–OOH) to maintain redox balance. Concurrently, iron uptake (via TFR), storage (ferritin), and release (mediated by NCOA4 and STEAP3) dynamically regulate labile Fe^2+^ levels. Excess Fe^2+^ drives Fenton reactions, generating reactive oxygen species (ROS) that oxidize polyunsaturated fatty acids (PUFAs) into cytotoxic lipid peroxides. The collapse of GPX4 activity and unchecked lipid peroxidation converge to trigger membrane destabilization and ferroptosis.

Therefore, autophagy is commonly regarded as a regulator of cell death [13]. Distinct from apoptosis, necroptosis, and pyroptosis, ferroptosis represent an iron-dependent form of cell death. At the cellular level, upregulated transferrin receptor 1 (TfR1)/divalent metal transporter 1 (DMT1)-mediated iron uptake, impaired ferroportin-mediated iron export, and ferritinophagy contribute to the expansion of the labile iron pool (LIP). This redox-active iron pool catalyzes the generation of lipid reactive oxygen species (ROS) through Fenton reaction, where Fe^2+^ reacts with hydrogen peroxide (H_2_O_2_) to produce hydroxyl radicals (•OH), initiating peroxidation of polyunsaturated fatty acids (PUFAs) in membrane phospholipids. The resulting lipid hydroperoxides, if not neutralized by glutathione peroxidase 4 (GPX4), compromise membrane integrity and trigger ferroptosis (Fig. 1) [[14], [15], [16], [17], [18], [19], [20]]. Previous studies have indicated that the development of various cardiovascular diseases is propelled by ferroptosis, including myocardial infarction (MI), heart failure (HF), and doxorubicin-induced cardiotoxicity (DIC) [[21], [22], [23]]. However, the interaction between ferroptosis and autophagy complicates therapeutic and intervention strategies due to their overlapping regulatory networks [24]. In this review, we take the interaction of autophagy and ferroptosis as an entry point and summarize their crosstalk, co-regulatory factors, pathophysiological mechanisms in various cardiovascular diseases, and potential therapeutic strategies. To the best of our knowledge, this is the inaugural review focusing on autophagy-ferroptosis interactions in CVDs, offering novel targets and deeper insight for the clinical treatment of CVDs.

## 2 Crosstalk between autophagy and ferroptosis

**Fig. 2:**
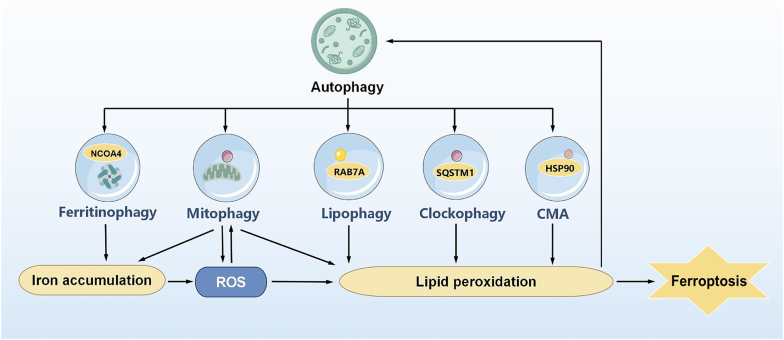
Crosstalk between autophagy and ferroptosis. Ferritinophagy, lipophagy, mitophagy, clockophagy, and chaperone-mediated autophagy (CMA) can regulate the ferroptosis process by modulating iron accumulation, ROS, and lipid peroxidation. Conversely, ROS and lipid peroxidation during ferroptosis, may reciprocally influence autophagic activity.

The intricate crosstalk between autophagy and ferroptosis represents a pivotal aspect of cellular homeostasis and disease pathogenesis. This intricate interplay involves multiple specialized forms of autophagy, including ferritinophagy, lipophagy, mitophagy, clockophagy, and chaperone-mediated autophagy (CMA), each exerting distinct yet interconnected effects on cellular iron metabolism, lipid homeostasis, and oxidative stress responses (Fig. 2). In this section, we comprehensively review the recent advancements in understanding the crosstalk between autophagy and ferroptosis.

### 2.1 Ferritinophagy

**Fig. 3:**
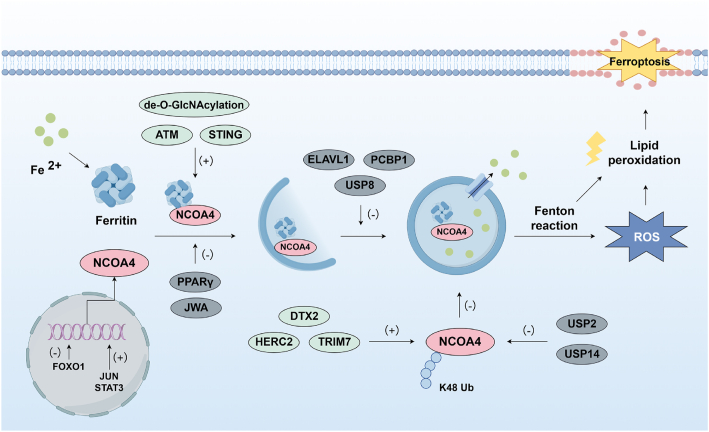
Ferritinophagy in ferroptosis. FOXO1 can inhibit the expression of NCOA4, while STAT3 and JUN promote the expression of NCOA4. ATM, STING and de-*O*-GlcNAcylation stimulate NCOA4-mediated autophagy-dependent ferroptosis, while JWA and prevent PPARγ the interaction between ferritin and NCOA4. HERC2, TRIM7 and DTX2 inhibit autophagy-dependent ferroptosis by promoting the ubiquitination degradation of NCOA4, while USP2 and USP14 maintains the stability of NCOA4 by counteracting its ubiquitination. ELAVL1, PCBP1, and USP8 inhibit autophagy activation to suppress ferritinophagy.

The progress of ferritinophagy involves the degradation of ferritin through the autophagy pathway, a crucial process for maintaining intracellular iron homeostasis. Ferritin is a cytosolic iron storage protein consisting of 24 subunits of ferritin heavy chain 1 (FTH1) and ferritin light chain (FTL) with a nanocage structure that can store more than 4500 iron atoms, and whose abundance and activity modulate intracellular iron bioavailability [25]. In this process, nuclear receptor coactivator 4 (NCOA4) functions as a cargo receptor, binding to ferritin and subsequently being recognized and degraded by lysosomes (Fig. 3). This process may be mediated by the binding of the C-terminus of NCOA4 to the conserved surface arginine (R23) on FTH1 in the phagophore [25,26]. Excess free iron released via ferritinophagy catalyzes the Fenton reaction, converting H_2_O_2_ into •OH, a highly reactive oxygen species. These radicals initiate iron-dependent lipid peroxidation by attacking PUFAs in cellular membranes, ultimately driving ferroptosis [27]. Conversely, the depletion of NCOA4 or autophagy-related gene (ATG) protein inhibited the ferroptosis [28,29]. These studies elucidated the relationship between NCOA4-dependent ferritinophagy and ferroptosis.

The intricate regulation of ferritinophagy, a pivotal cellular process essential for maintaining intracellular iron homeostasis, is governed by a complex interplay of molecular mechanisms. At the transcriptional level, forkhead box transcription factor O1 (FOXO1) has been identified as a transcriptional repressor of NCOA4 to directly suppress NCOA4 expression [30]. Conversely, signal transducer and activator of transcription 3 (STAT3) and JUN have been revealed to promote NCOA4 expression [31,32]. In addition to transcriptional control, the stability of NCOA4 is dynamically modulated by ubiquitination processes. Extensive researches demonstrates that HECT and RLD domain-containing E3 ubiquitin protein ligase 2 (HERC2), E3 ubiquitin ligase deltex 2 (DTX2), and tripartite motif-containing protein 7 (TRIM7) function as E3 ubiquitin ligases that promote NCOA4 degradation, thereby suppressing NCOA4-driven ferritinophagy and ferroptosis [[33], [34], [35]]. Conversely, ubiquitin-specific protease 14 (USP14) and ubiquitin-specific protease 2 (USP2) have been reported to stabilize NCOA4 by de-ubiquitination, triggering ferritinophagy and ferroptosis [36,37]. From another perspective, mechanical tension has been observed to modulate NCOA4-dependent ferritinophagy, with reduced tension decreasing free iron levels via enhanced FTH1 expression and suppressed NCOA4 activity [38].

Another critical regulatory node in this process is the NCOA4-ferritin interaction. Wu et al. revealed that the DNA damage sensor ataxia telangiectasia mutated (ATM) kinase facilitates their interaction via NCOA4 phosphorylation, thereby sustaining ferritinophagy [39]. Moreover, stimulator of interferon genes (STING) has been demonstrated to interact with NCOA4 through key binding residues (Q237, E316, S322) in its CBD domain, which triggers ferritinophagy-mediated ferroptosis while stabilizing STING dimers to amplify inflammatory responses [40,41]. In addition to NCOA4, O-GlcNAcylation removal at S179 of ferritin heavy chain have been showed to enhance its binding to the ferritinophagy receptor NCOA4 [42]. On the other hand, evidence suggests that Poly-ADP-ribose polymerases (PPARγ) competitively blocks NCOA4-ferritin binding through interaction with NCOA4's LxxLL motif in vascular smooth muscle cell senescence and arterial aging [43]. Similarly, JWA has been showed to alleviate ferroptosis in dopamine neurons by occupying NCOA4-ferritin binding site [44].

Autophagosome formation and degradation also critically regulate ferritinophagy. It has been reported that transmembrane protein 164 (TMEM164) selectively mediates ATG5-dependent autophagosome formation during ferritinophagy-mediated ferroptosis, distinct from starvation-induced autophagy [45,46]. Conversely, Liu et al. demonstrated that ubiquitin-specific protease 8 (USP8) suppresses ferritinophagy by de-ubiquitinating sequestosome 1 (SQSTM1/p62) and impairing its autophagy receptor function [47]. Besides, upregulated ELAV like RNA binding protein 1 (ELAVL1) and Poly(rC)-binding protein 1(PCBP1) are revealed to inhibit autophagy activation through binding to CU-rich elements in Beclin-1 (BECN1) mRNA 3′-UTR, consequently suppressing ferritinophagy [45,46,48]. Furthermore, emerging evidence suggests that mitochondrial ROS (mtROS) participate in ferritinophagy regulation by contributing to autophagy activation. Mechanistically, mtROS has been shown to induce AMP-activated protein kinase (AMPK) phosphorylation at Thr172, which subsequently modulates unc-51-like autophagy activating kinase 1 (ULK1) phosphorylation at Ser555, thereby driving ferritinophagy [[49], [50], [51]]. Beyond this, mtROS accumulation is also observed to promote endoplasmic reticulum (ER) stress, activating protein kinase RNA-like ER kinase (PERK)-eukaryotic initiation factor 2 α subunit (eIF2α)- activating transcription factor 4 (ATF4)- C/EBP homologous protein (CHOP) signaling to promote ferritinophagy [52]. Moreover, Ironomycin (AM5), a salinomycin derivative, has been revealed to kill cancer stem cells by accumulating and chelating iron in lysosomes, depleting cytoplasmic iron, and triggering ferritinophagy to induce ferroptosis [53]. Interestingly, existing studies indicate that salinomycin prevents fibrosis and pathological cardiac remodeling [54], as well as enhances myocardial blood perfusion and mechanical efficiency [55]. Conversely, it is also shown that high-dose salinomycin can lead to arrhythmia and myocardial death [56,57]. These effects are likely cell-specific and dose-dependent, requiring further investigation.

### 2.2 Lipophagy

**Fig. 4:**
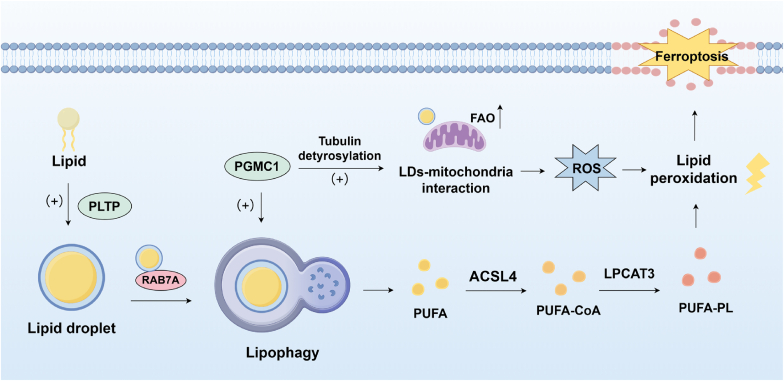
Lipophagy in ferroptosis. Lipophagy, the process of autophagic degradation of lipid droplets—specialized organelles that store neutral lipids. RAB7A, a protein associated with lipid droplets, facilitates lipophagy and promotes ferroptosis. In contrast, lipid droplet formation mediated by PLTP serves as a protective mechanism, inhibiting lipophagy-dependent ferroptosis. Meanwhile, PGRMC1 enhances ferroptosis by inducing lipophagy.

Lipophagy represents a fundamental cellular mechanism characterized by the precise recognition and subsequent degradation of lipid droplets (LDs), playing a pivotal role in maintaining intracellular lipid metabolic homeostasis (Fig. 4). LDs, serving as primary storage sites for neutral lipids such as triacylglycerol (TAG) and sterol esters, are ubiquitously present across diverse organisms ranging from bacteria to humans. During lipophagy, lysosomal acid lipases hydrolyze neutral lipids within LDs into free fatty acids (FFAs), unesterified cholesterol, and glycerol as primary degradation products. These FFAs not only undergo β-oxidation for adenosine triphosphate (ATP) generation but also function as precursors for bioactive lipid mediators. Specifically, fatty acids act as signaling ligands to activate peroxisome proliferator-activated receptor gamma coactivator 1α (PGC1α) and/or peroxisome proliferator-activated receptor α (PPARα)-dependent transcriptional programs governing lipophagy gene expression [58]. Additionally, they serve as allosteric modulators and metabolic substrates for generating secondary messengers including eicosanoids and sphingolipids. In the context of ferroptosis, Bai et al. demonstrated for the first time that autophagic degradation of intracellular lipid droplets promotes RSL3-induced hepatocyte ferroptosis, primarily relying on autophagy-related proteins RAB7A [59,60]. Mechanistically, Yu et al. revealed that PUFAs generated via lipophagy undergo lipid peroxidation through Acyl-CoA synthetase long-chain family member 4 (ACSL4) and lysophosphatidylcholine acyltransferase 3 (LPCAT3) activity, contributing to di(2-ethylhexyl) phthalate (DEHP)-induced renal ferroptosis [61]. Similarly, ACSL4 has been identified to facilitate lipid peroxidation of FFAs released post-lipophagy in sepsis-induced acute kidney injury [60,62]. Furthermore, lipophagy-generated FFAs have been demonstrated to be processed through mitochondrial oxidation, enhancing fatty acid oxidation (FAO) and generating ROS that exacerbate lipid peroxidation [63,64]. Crucially, excessive accumulation of FFAs and ROS has been shown to interfere with normal mitochondrial function, which further exacerbates the accumulation of FFAs released through lipophagy, thereby creating a vicious cycle that ultimately drives to ferroptosis [63,64]. In cardiovascular research, Wang et al. monitored LDs dynamics in H9c2 cardiomyocytes during ischemia-reperfusion injury using aggregation-induced emission (AIE)-based probes. Their results revealed an initial increase in LDs quantity during the early phase, followed by lipophagy-mediated reduction at later stages. Notably, the reduction of LDs decomposition significantly attenuates lipid peroxidation levels in cardiomyocytes [59].

Lipophagy regulation involves additional molecules with therapeutic potential. It has been reported that progesterone receptor membrane component 1 (PGRMC1) modulates SIRT1 expression to promote AMPK phosphorylation, thereby enhancing lipophagy and increasing sensitivity to ferroptosis inducers [63]. Beyond this, it also enhances LDs-mitochondria interactions by facilitating microtubule detyrosination, synergizing FAO with lipophagic processes [63]. Conversely, deletion of phospholipid transfer protein (PLTP), a p53 target gene has been demonstrated to markedly increase cellular sensitivity to ferroptosis inducers, while its overexpression promotes intracellular LDs formation, suggesting it may attenuate ferroptosis by facilitating lipid storage [65]. Furthermore, numerous studies have shown that macrophage lipophagy plays a crucial role in promoting cholesterol efflux to combat atherosclerosis development [66]. Recent research indicates that 7-dehydrocholesterol (7-DHC), a cholesterol precursor, inhibits ferroptosis by preferentially reacting with peroxyl radicals to block phospholipid peroxidation [67]. Future studies may need to further explore the role of cholesterol metabolism in the interaction between lipophagy and ferroptosis during cardiovascular diseases.

### 2.3 Mitophagy

**Fig. 5:**
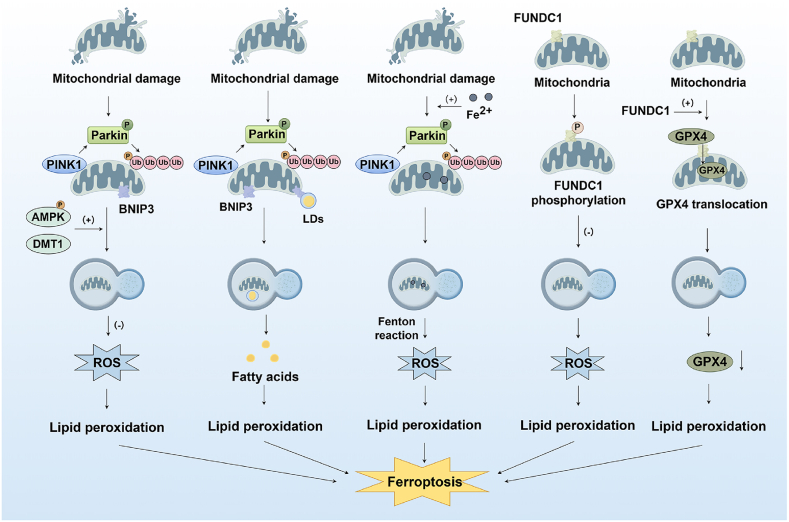
Mitophagy in ferroptosis. Phosphorylation-mediated activation of AMP-activated protein kinase (AMPK) and divalent metal transporter 1 (DMT1) promotes PTEN-induced putative kinase 1 (PINK1)/Parkin RBR E3 ubiquitin protein ligase (Parkin)-dependent mitophagy, thereby reducing ROS generation and suppressing ferroptosis. Under radiation exposure, the contact between lipid droplets and mitochondria increases, and mitophagy generates excessive fatty acids that promote lipid peroxidation. PM2.5 induces intracellular Fe^2+^ accumulation, which are subsequently translocated into mitochondria. During mitophagy, mitochondrial iron is released into the cytosol, further promoting ferroptosis. FUN14 domain-containing 1 (FUNDC1), a key mitophagy receptor, mediates mitochondrial clearance. FUNDC1 phosphorylation inhibits mitophagy, resulting in mitochondrial dysfunction and excessive ROS production. Additionally, FUNDC1 recruits and binds to GPX4, facilitating its degradation via mitophagy to drive ferroptosis.

Mitophagy is a selective autophagy process through which cells specifically recognize and degrade damaged or dysfunctional mitochondria via ubiquitin-dependent pathways (e.g., PINK1/Parkin) or ubiquitin-independent receptor-mediated mechanisms (e.g., BNIP3/NIX/FUNDC1), thereby maintaining mitochondrial quality and intracellular homeostasis (Fig. 5). Emerging evidence has unveiled a multifaceted relationship between mitophagy and ferroptosis, where their interactions are context-dependent and modulated by intricate signaling networks [68]. Moderate mitophagy suppresses ferroptosis by eliminating dysfunctional mitochondria, reducing mtROS release and Fe^2+^ efflux, thereby attenuating lipid peroxidation. Specifically, key sources of mtROS include electron leakage at complexes I and III of the, impaired electron transport chain, generating superoxide radicals [69] and iron overload within retained mitochondria, which amplifies Fenton reactions [70]. For instance, in cisplatin-induced renal injury, B-cell lymphoma 2 (Bcl-2)/adenovirus E1B 19 kDa protein-interacting protein 3 (BNIP3) or PTEN-induced putative kinase 1 (PINK1)/Parkin RBR E3 ubiquitin protein ligase (Parkin) mediated mitophagy significantly alleviates ferroptosis through degradation of damaged mitochondria, which reduces oxidative stress and iron accumulation [71]. Conversely, excessive mitophagy may degrade ferritin via lysosomes, releasing free iron into the LIP and exacerbating Fenton reactions and lipid peroxidation to promote ferroptosis. This dual role is determined by cellular stress levels and the dynamic equilibrium of mitophagy.

The ubiquitin-dependent PINK1/Parkin mitophagy pathway represents the most extensively studied mechanism of mitophagy [72]. PINK1, functioning as a mitochondrial damage sensor, stabilizes on the outer mitochondrial membrane (OMM) upon depolarization and phosphorylates ubiquitin and Parkin, thereby activating the latter E3 ubiquitin ligase activity. Parkin subsequently ubiquitinates mitochondrial surface proteins, forming ubiquitin chains that recruit autophagy receptors for microtubule-associated protein 1 light chain 3 (LC3) binding, ultimately leading to autophagosome formation. Studies have demonstrated that melatonin enhances PINK1-Parkin-regulated mitophagy by promoting AMPK phosphorylation, which reduces excessive ROS release and suppresses ferroptosis [73,74]. Beyond this, in nicorandil-treated models, AMPK phosphorylation has also been shown to inhibit ACSL4 translocation to mitochondria, thereby suppressing mitochondria-associated ferroptosis [73,74]. Additionally, emerging evidence indicates that the modulation of mitochondrial complex activities impacts mitophagy-dependent ferroptosis. Inhibition of mitochondrial complex I by BAY 87–2243 has been shown to trigger mitophagy-dependent ROS accumulation, leading to necroptosis and ferroptosis [75,76]. Furthermore, DMT1 has been identified as a key regulator of mitochondrial membrane potential, and its deficiency increases mitochondrial complex I activity to synergistically suppress Erastin-induced ferroptosis [75,76]. Moreover, several studies indicate that external environmental stimuli can trigger mitophagy, thereby promoting ferroptosis. Ionizing radiation, a common clinical treatment for eliminating malignant tumors, has been shown to induce excessive mitochondrial ROS production and mitochondrial damage, while concurrently causing lipid droplets to accumulate around damaged mitochondria to redistribute fatty acids. Simultaneously, mitophagy is activated via the PINK1/Parkin and BNIP3 pathway, releasing FFAs into the cytoplasm. These fatty acids undergo ROS-mediated peroxidation, further driving ferroptosis [77]. Beyond ionizing radiation, PM2.5 exposure also promotes ferroptosis through mitophagy. PM2.5 induces heme oxygenase-1 (HO-1) upregulation in hippocampal neurons, leading to increased iron release and disrupted iron metabolism. Excessive iron ions are transported into mitochondria, causing mitochondrial damage and activating PINK1/Parkin-mediated mitophagy. This mitophagy process provides an additional iron source for ferroptosis, exacerbating its progression [78]. These studies have revealed the close association between mitophagy and Fe^2+^/lipid droplets, and also suggest that the complex interplay mechanisms among mitophagy, ferritinophagy, and lipophagy may require further exploration.

FUN14 domain-containing 1 (FUNDC1), a mitophagy receptor, mediates ubiquitin-independent mitophagy through its interaction with the LC3-interacting region of LC3. Studies have demonstrated that FUNDC1 interacts with GPX4 to facilitate its translocation into mitochondria via the TOM/TIM complexes, where GPX4 is degraded through mitophagy, thereby triggering ferroptosis [79]. Furthermore, FUNDC1 phosphorylation has been revealed to inhibit mitophagy, leading to mitochondrial dysfunction, excessive ROS generation, and ferroptosis in intestinal endothelial cells [80]. Additionally, FUNDC1 deficiency has been implicated in sensitizing cells to ferroptosis under high-fat diet exposure, highlighting its crucial role in maintaining mitochondrial health and preventing ferroptosis [81]. Beyond FUNDC1, BNIP3 also functions as a mitophagy receptor that mediates ubiquitin-independent mitophagy. Studies have demonstrated that the BNIP3/NIP3-like protein X (NIX) mediates mitophagy, which effectively reduces mtROS levels, thereby protecting cells from ferroptosis [56]. Additionally, BNIP3-mediated mitophagy has been reported to drive the p62-Keap1-Nrf2 pathway, which maintains iron and redox homeostasis by regulating FTH1 and heme oxygenase-1, thereby counteracting ferroptosis [57].What's more, short-chain fatty acids (SCFAs) have been observed to upregulate NOD-like receptor family pyrin domain-containing 6 (Nlrp6) expression, which in turn enhances retinoic acid-inducible gene I (RIG-1)/mitochondrial antiviral signaling proteins (MAVS)-mediated mitophagy, preventing ferroptosis [82].

### 2.4 Clockophagy

**Fig. 6:**
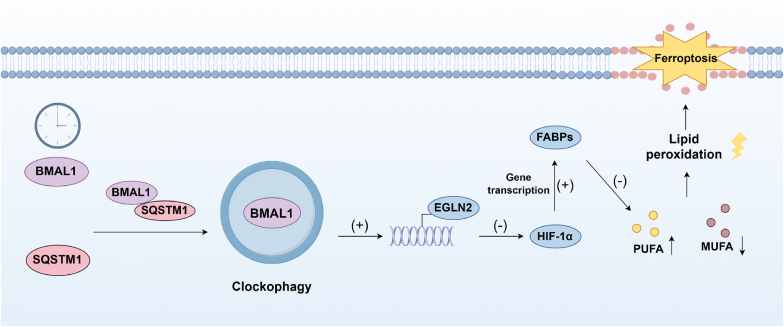
Clockophagy in ferroptosis. Core clock transcription factor brain and muscle ARNT-like 1 (BMAL1), a key transcription factor in the circadian clock, is selectively degraded via autophagy in a process termed clockophagy. The autophagy receptor SQSTM1 mediates the recognition and degradation of BMAL1 during this process. BMAL1 degradation leads to the upregulation of its target gene, hypoxia-inducible factor 2 of the egl-9 family (EGLN2), which subsequently suppresses the activity of hypoxia-inducible factor-1α (HIF-1α). HIF-1α promotes the expression of FABP3 and FABP7, proteins involved in fatty acid uptake and lipid storage. By limiting the availability of polyunsaturated fatty acids (PUFAs) and driving monounsaturated fatty acids (MUFAs) accumulation, these proteins influence ferroptosis susceptibility.

Clockophagy, a proposed selective autophagy process, has been reported to mediate the degradation of the core circadian clock transcription factor brain and muscle ARNT-like 1 (BMAL1) during ferroptosis induced by type 2 activators (RSL3 and FIN56) (Fig. 6) [83,84]. These works suggest that SQSTM1/p62 may act as an adaptor for BMAL1 recognition in this context (Fig. 6). However, it is noteworthy that this phenomenon appears to be stimulus-specific, as BMAL1 degradation was not observed in ferroptosis triggered by SLC7A11 inhibitors (e.g., erastin, sulfasalazine) [83,84]. This may occur because different ferroptosis activators trigger distinct signaling pathways, and the exact molecular mechanisms require further investigation. As a fundamental component of the circadian clock system, BMAL1 sustains the circadian rhythm of organisms by orchestrating the rhythmic expression of genes, including its inhibitors PER and CRA [85]. The degradation of BMAL1 significantly impacts the occurrence of lipid peroxidation and ferroptosis. Mechanistically, the degradation of BMAL1 has been demonstrated to lead to the upregulation of its target gene hypoxia-inducible factor 2 of the egl-9 family (EGLN2), which subsequently inhibits the function of hypoxia-inducible factor-1α (HIF-1α) [86]. HIF-1α has been identified to facilitate fatty acid uptake and lipid storage by inducing fatty acid-binding proteins 3 and 7 (FABP3/7) [83,84,87]. Emerging mechanistic insights reveal that FABP3 mediates PUFAs metabolism through circRNA_101093 interaction, facilitating N-arachidonoyl taurine (NAT) production that limits PUFA membrane incorporation and ferroptosis susceptibility [88]. Conversely, FABP7 has been observed to drive monounsaturated fatty acids (MUFA) accumulation and triglyceride synthesis, effectively suppressing lipid peroxidation and ROS formation [89]. Additionally, evidence suggests that BMAL1 also plays a pivotal role in regulating the expression of various antioxidant or membrane repair systems, conferring protection against ferroptosis [86]. Importantly, the concept of circadian-autophagy crosstalk in ferroptosis regulation is emerging but not yet universally established. It is essential to conduct rigorous mechanistic exploration and independent validation of the clockophagy mediated-ferroptosis hypothesis across various cell types.

### 2.5 Chaperone-mediated autophagy (CMA)

**Fig. 7:**
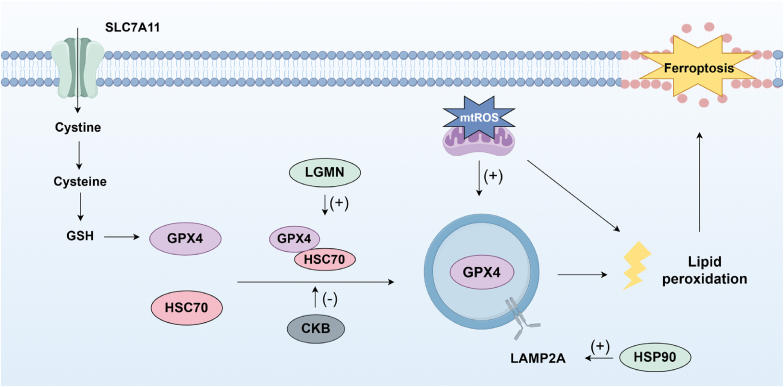
Chaperone-mediated autophagy (CMA) plays a critical role in the degradation of glutathione peroxidase 4 (GPX4) during ferroptosis. Lysosome-associated membrane protein type 2A (LAMP2A) facilitates the translocation of GPX4 across the lysosomal membrane, while heat shock cognate protein 70 (HSC70) interacts with both GPX4 and LAMP2 to enable this process. The heat shock protein (HSP90) enhances the protein stability of the CMA receptor LAMP2A. The CMA-mediated degradation of GPX4 is negatively regulated by creatine kinase B (CKB)-mediated phosphorylation of GPX4. Additionally, Legumain (LGMN) supports the CMA-dependent degradation of GPX4. Mitochondrial reactive oxygen species (mtROS) further contribute to GPX4 degradation, highlighting their involvement in ferroptosis.

CMA represents a highly selective autophagic process that recognizes and binds proteins containing KFERQ motifs directly through chaperones, subsequently transporting these proteins to lysosomes for degradation. Previous studies have extensively demonstrated that CMA drives ferroptosis under diverse stress conditions through GPX4 degradation, a process primarily mediated by the chaperone heat shock cognate protein 70 (HSC70) and lysosome-associated membrane protein type 2A (LAMP2A) (Fig. 7) [90]. For example, chemical toxins including antimon [90,91], fluorid [92], BDE-4 [93], and erastin [94] promote CMA-dependent GPX4 proteolysis, while pathogens such as Mycobacterium bovis exploit this pathway through Mb3523c-mediated heat shock protein (HSP) 90 hijacking [95]. Furthermore, oxidative insults such as H_2_O_2_ trigger HSP90 cleavage, which amplifies CMA-mediated GPX4 degradation. This process is effectively reversed by quercetin through HSP90 stabilization (Fig. 7) [96].

The CMA process is regulated by multiple molecules and may serve as a potential therapeutic target. Notably, the HSP90 has been demonstrated to enhance the protein stability of the CMA receptor LAMP2A, ultimately augmenting GPX4 degradation and promoting ferroptosis [94]. Legumain (LGMN) an asparagine endopeptidase, has been shown to facilitate chaperone-mediated GPX4 autophagy by interacting with HSC70, HSP90, and GPX4 [97]. Previous studies have established that oxidative stress induces oxidative modifications of CMA substrate proteins, significantly enhancing their binding affinity to chaperones while upregulating the expression of lysosomal membrane protein LAMP2A. These coordinated mechanisms facilitate substrate recognition and degradation through the CMA pathway [96,98,99]. Consistently, emerging evidence demonstrates oxidative stress-mediated control of GPX4 degradation via CMA. Mechanistically, Hirata et al. revealed that conjugated fatty acids (CFAs) utilize CPT1/2 transporters for mitochondrial entry, where ACSL1-dependent generation of mtROS and lipid peroxides activates CMA-mediated lysosomal degradation of GPX4 [100]. Besides, antimony induced ROS has been reported to inhibit Transient receptor potential mucolipin 1 (TRPML1) expression, which disrupts its negative regulation of CMA, ultimately driving ferroptosis through enhanced GPX4 degradation [91]. The ROS may derive from mitochondrial sources, cytosolic oxidase systems, or other alternative cellular compartments. Substantiating the mitochondrial contribution, mitochondria-targeted antioxidant TEMPO was shown to suppress LAMP2A/HSP70 upregulation while restoring GPX4 levels and alleviating ferroptosis [92]. These collective findings establish ROS as pivotal initiators of CMA-dependent GPX4 degradation, though precise regulatory mechanisms need further elucidation. Conversely, the phosphorylation of creatine kinase B (CKB) has been demonstrated to increase its binding affinity to GPX4 and phosphorylates GPX4 at S104, thereby preventing the binding of HSC70 to GPX4 [101]. In addition to GPX4, ACSL4 can be recognized by the CMA receptor HSC70 and is ultimately degraded within lysosomes. Abnormalities in the CMA process result in ACSL4 accumulation, which promotes the generation of lethal lipid peroxides and subsequently induces ferroptosis in retinal pigment epithelial (RPE) cells [102].

### 2.6 Others

**Fig. 8:**
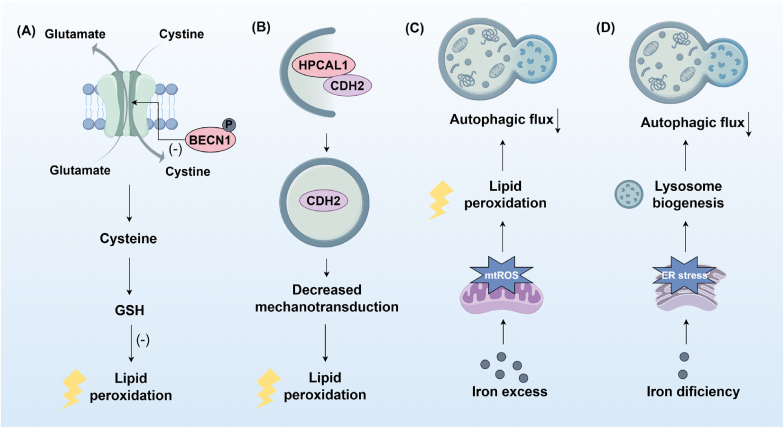
Other crosstalk between ferroptosis and autophagy. Phosphorylated CU-rich elements in Beclin-1 (BECN1) interacts with SLC7A11 to inhibit system Xc-, reducing intracellular GSH levels and promoting lipid peroxidation. Similarly, hippocalcin like 1 (HPCAL1)-mediated phosphorylation of cadherin 2 (CDH2) decreases mechano-transduction, further driving lipid peroxidation. Iron excess elevates mitochondrial ROS (mtROS), enhancing lipid peroxidation and stimulating autophagic flux. In contrast, iron deficiency leads to endoplasmic reticulum (ER), which impairs lysosome biogenesis and reduces autophagic flux, highlighting the dual role of autophagy in regulating ferroptosis susceptibility.

The intricate interplay between ferroptosis and autophagy, particularly in the context of the cystine/glutamate antiporter System Xc- and GSH homeostasis, has garnered significant attention. System Xc-, a pivotal component of cellular antioxidant defenses, orchestrates the influx of cystine, the oxidized counterpart of cysteine, in exchange for glutamate. This biochemical exchange fuels the intracellular reduction of cystine to cysteine, a rate-limiting precursor in GSH synthesis, thus maintaining GSH homeostasis and mitigating oxidative stress. Recent advancements in the field have elucidated novel regulatory mechanisms linking autophagy with System Xc-activity and GSH levels. Specifically, autophagy-associated proteins, notably Beclin-1, have emerged as direct regulators of System Xc-, binding to and inhibiting its function, thereby influencing GSH levels and ferroptosis sensitivity (Fig. 8) [103,104]. This intricate crosstalk underscores the active participation of autophagy in shaping the cellular redox milieu by modulating key determinants of GSH metabolism. Furthermore, the recent identification of hippocalcin like 1 (HPCAL1) as a distinct autophagy receptor intricately entwined in ferroptosis. By facilitating the selective degradation of cadherin 2 (CDH2), HPCAL1 dynamically modulates membrane tension and thereby augments susceptibility to ferroptosis (Fig. 8) [105]. Besides, recent studies have highlighted the critical role of iron in modulating autophagy. Excess iron increases mtROS production, which amplifies lipid peroxidation and enhances autophagic flux, thereby promoting ferroptosis [106]. In contrast, iron deficiency triggers ER stress, leading to impaired lysosome biogenesis and reduced autophagic flux [106,107]. During ferroptosis, ROS and lipid peroxidation cooperatively regulate autophagy through multiple molecular pathways. Experimental evidence indicates that ROS generated during ferroptosis activates the AMPK pathway, which phosphorylates and activates the ULK1 complex to initiate autophagosome formation [108]. Besides, ROS has been showed to stimulate the Nrf2 pathway, triggering protective autophagy as a compensatory response to ferroptotic stress [109]. The mitogen-activated protein kinase (MAPK) pathway is another ROS-sensitive cascade that activates transcription factor EB (TFEB), enhancing lysosomal biogenesis and autophagy-related gene expression [110]. Additionally, ROS-dependent p53 signaling upregulates Beclin1, a critical autophagy-initiating protein [111]. Lipid peroxidation products, including 4-hydroxynonenal, activate c-Jun N-terminal kinase (JNK)-mediated autophagy [112], while SIN-1-induced lipid peroxidation facilitates TP53INP1 interaction with LC3 proteins to drive autophagosome maturation [113,114]. These findings collectively demonstrate that ROS and lipid peroxidation serve as both initiators and amplifiers of autophagy during ferroptosis, and underscore the crosstalk between ferroptosis and autophagy.

## 3 Co-regulators of autophagy and ferroptosis

**Fig. 9:**
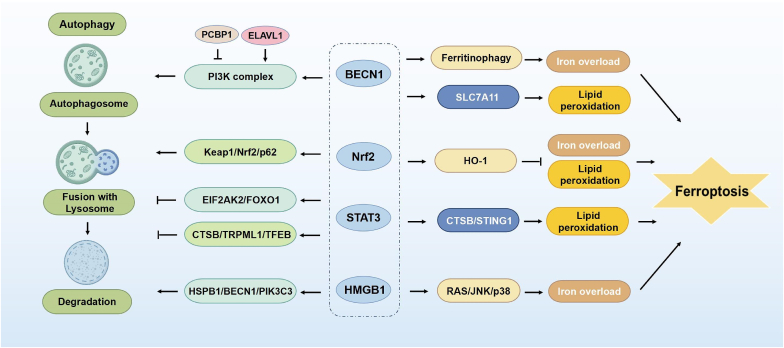
Co-regulators of autophagy and ferroptosis. Beclin-1 triggers ferroptosis by enhancing ferritin autophagy flux or inhibiting system Xc-activity. Nrf2 promotes autophagy and suppresses ferroptosis through the Keap1/Nrf2/p62 positive feedback loop and the Nrf2-HO-1 axis. The STAT3/CTSB axis stimulates autophagy-dependent ferroptosis by permeabilizing lysosomal membranes. Additionally, HMGB1 induces autophagy through various pathways depending on its subcellular location and promotes ferroptosis by stimulating the downstream RAS/JNK/p38 pathway.

Recently, growing evidence has demonstrated that the interaction between autophagy and ferroptosis is intimately associated with key co-regulators such as Beclin-1, Nrf2, STAT3, and high mobility group box 1 (HMGB1), with various signaling pathways interconnecting to form a complex regulatory network. This review summarizes the essential signaling molecules and pathways involved in the crosstalk of autophagy and ferroptosis (Fig. 9).

### 3.1 Beclin-1

Beclin-1, alternatively known as ATG6, is the first identified autophagy-related protein in mammals and essential for initiating and regulating autophagy. It is vital for cellular metabolism, homeostasis, and disease development [115,116]. Beclin-1 is a molecular scaffold for the phosphatidylinositol-3-kinase (PI3K) complex, which triggers autophagosome formation. It also plays a crucial role in autophagosome maturation and lysosomal fusion by interacting with various autophagy-associated proteins, such as ultraviolet radiation resistance-associated gene (UVRAG) and ATG5-interacting membrane-bound protein 1 (Ambra1), thereby regulating autophagic flux [117,118]. Phosphorylation and ubiquitination could modulate Beclin-1-dependent autophagy levels and involve various physiological processes [119]. In response to nutritional stress, ULK1 promotes autophagy by phosphorylating Beclin-1 at the N-terminal Ser14, 15, or Ser30 sites, which enhances its interaction with vacuolar protein sorting 34 (Vps34), thus supplying substrate and energy [120]. Enhanced interaction of Bcl-2 with Beclin-1's Bcl-2 homology-3 (BH3) domain, and the administration of the autophagy-inducing peptide Tat-Beclin1 (TB), have been demonstrated to stimulate autophagic flux and preserve mitochondrial homeostasis [116,121]. Furthermore, E3 ubiquitin ligase Cullin3 (CUL3) and other proteins could suppress autophagy by degrading Beclin-1 [119].

Recent studies have indicated that autophagy regulators, including Beclin-1, cathepsin B (CTSB), HMGB1, and mammalian target of rapamycin (mTOR), are crucial in regulating ferroptosis [17]. Notably, Beclin-1 performs a conventional function in autophagy by enhancing ferritinophagic flux, leading to intracellular free iron overload, and inducing ferroptosis. It is established that ELAVL1 could be bound to the AU-rich element (ARE) in the 3′-untranslated region (3′-UTR) of Beclin-1 mRNA and enhancing its stability, thereby triggering ferritinophagy-mediated ferroptosis. Conversely, decreasing autophagy or deleting the F3 region could inhibit ELAVL1-mediated ferroptosis [48]. Cytoplasmic iron chaperone poly (rC)-binding protein 1 (PCBP1) could prevent ferroptosis by binding to CU-rich elements in the 3′-UTR of Beclin-1 mRNA, driving its degradation [45]. USP11 has been demonstrated to enhance ferroptosis by activating autophagy through the deubiquitination of Beclin-1 [122].

Additionally, Beclin-1 presents great potential to mediate ferroptosis through alternative pathways. Literature indicates that in the absence of Beclin-1-PtdIns3K complex formation, AMPK could promote the phosphorylation of Belcin-1 at Ser90/93/96, resulting in the formation of a complex with the cystine/glutamate reverse transporter protein (SLC7A11). This complex inhibits the activity of the system Xc-transporter, resulting in intracellular GSH depletion and impairing GPX4 in utilizing GSH to reduce lipid hydroperoxides to lipids, ultimately triggering classical ferroptosis [27,103,123]. This pathway promotes lipid peroxidation without influencing intracellular iron accumulation or metabolism [123]. Beclin-1 knockdown inhibited ferroptosis triggered by type 1 activators including Erastin, SAS, and sorafenib, but did not affect other activators, such as RSL3, FIN56, and bupivacaine sulfoximine [25,103,124]. Another study demonstrated that human umbilical cord mesenchymal stem cells (hucMSCs)-derived exosomes elevate BECN1, which inhibits system xc-activity and GPX4 expression by interacting with SLC7A11, ultimately inducing ferroptosis [125].

### 3.2 Nrf2

Nrf2 is a crucial transcription factor that modulates cellular responses to oxidative stress and inflammation. In the resting state, Nrf2 is anchored in the cytoplasm by binding to Kelch-like ECH-associated protein 1 (Keap1), where it is rapidly degraded through the CUL3-mediated ubiquitin-proteasome pathway to maintain low levels [17]. In response to environmental stressors such as oxidative and electrophilic stress, Keap1 undergoes deactivation and dissociation from Nrf2, which is stabilized and translocated to the nucleus. This stabilization enables Nrf2 to translocate to the nucleus, where it forms heterodimers with small Maf proteins to bind specifically to antioxidant response elements in the promoter regions of target genes in a sequence-specific manner, therefore stimulating the transcription of cytoprotective genes [16,18]. Cells have developed several defense mechanisms against ferroptosis by detoxifying lipid hydroperoxides, including the GSH system, the coenzyme Q10 (CoQ10) system, and the thioredoxin system [126]. Numerous downstream genes of Nrf2 are involved in the ferroptosis process, including the regulation of intracellular GSH levels (glutathione S-transferase (Gst), SLC7A11, GSH reductase, GPX4, etc.), the regulation of NADPH levels to modulate the activity of GPX4 and coenzyme Q10 (glucose-6-phosphate dehydrogenase (G6PD), NAD(P)H quinone dehydrogenase 1 (NQO1), phosphogluconate dehydrogenase (PGD), etc.), regulation of ROS content and involvement in the thioredoxin (TXN) system (sulforaphane (Srxn1), thioredoxin reductase 1 (Txnrd1), etc.) [[16], [17], [18], [19], [20]] Therefore, it is widely acknowledged that Nrf2 has the potential to block ferroptosis through the inhibition of lipid peroxidation. In addition, Nrf2 exerts its modulatory effect on ferroptosis through the Nrf2/HO-1 axis. It enhances the activity of HO-1, the initiating and rate-limiting enzyme of heme degradation, and accelerates the conversion of iron-containing heme into CO, biliverdin, and ferrous ions [18]. CO and biliverdin have anti-oxidant potential, while nascent dissociative iron can be repurposed or stored by cells to avert iron overload [127]. It is established that Nrf2 activators, such as sulforaphane, dimethyl fumarate, proanthocyanidins, songorine, and bardoxolone methyl, have the capacity to exert mitochondrial protection, thereby influencing autophagy or ferroptosis [128]. Activation of the Nrf2/HO-1 axis has been observed to stimulate FTH1 and ferroportin, which are involved in the storage and transfer of excess iron. While suppressing iron uptake-related genes like DMT1 and hepcidin. This process results in a reduction of intracellular iron levels and inhibits ferroptosis [16,129]. However, excessive activation of HO-1 may lead to iron overload due to the inability to utilize free iron promptly, which in turn might trigger ferroptosis [130].

The regulation of Nrf2-associated autophagy has garnered significant attention. Besides indirectly enhancing autophagy by reducing oxidative stress, Nrf2 promotes the expression of various autophagy-related genes, such as ATG protein family members, lysosomal degradative enzymes, Beclin-1, and ULK1, to directly boost cellular autophagy [130,131]. The evidence suggests that the molecular mechanisms by which Nrf2 regulates autophagy are associated with ferroptosis [130]. The selective autophagy receptor p62 modulates autophagy by directing ubiquitinated proteins to the autophagosome for degradation [132]. When exposed to various types of ferroptosis activators, p62 promotes autophagic degradation of Keap1 to upregulate the Nrf2 level, and Nrf2 transactivates the SQSTM1 gene to promote p62 expression, forming a Keap1/Nrf2/p62 positive feedback loop with the potential to enhance autophagy and inhibit ferroptosis [133]. Additionally, the Keap1-Nrf2 pathway promotes FSP1, which suppresses lipid peroxidation and inhibits ferroptosis by reducing coenzyme Q10 at the plasma membrane and decreasing vitamin K and ROS levels [134,135]. Moreover, inhibition of the Nrf2/HO-1 Axis may promote the abundance of ferritin autophagy-associated proteins and autophagosomes by enhancing LC3 activity, ultimately contributing to ferroptosis event by iron overloading [136].

### 3.3 STAT3

Upon stimulation (hyperoside, interleukin-6 (IL-6), fibrinogen-like protein 1 (FGL1), silibinin, etc.), STAT3 undergoes self-activated and subsequent translocation to the nucleus to promote target gene transcription [[137], [138], [139], [140]]. STAT3 exerts position-dependent effects in the precise regulation of autophagy. Nuclear STAT3 primarily inhibits autophagy by mediating the transcription of autophagy-related genes, including CTSB, Bcl2 family members, Beclin-1, BNIP3, HIF-1α, and related microRNAs. Cytoplasmic STAT3 also inhibits autophagy by chelating eukaryotic translation initiation factor 2α kinase 2 (EIF2AK2), forkhead box O (FOXO) 1, and FOXO3, thereby exerting a constitutive suppression of autophagy [141,142]. STAT3 also inhibits autophagy via a lysosome-dependent mechanism. The phosphorylation of STAT3 at the Tyr705 site prevents TFEB from translocating to the nucleus and binding to precise-binding elements (PBATs) in the 3′-UTR of autophagy-related mRNAs, such as the ATG family. This blockage disrupts lysosomal membrane permeabilization (LMP) and the autophagy-lysosomal pathway [143]. The inhibitory effect of STAT3 on TFEB may involve the cleavage of the lysosomal calcium channel TRPML1 by the cysteine protease CTSB. This inhibition of calcium efflux reduces the activity of the serine/threonine phosphatase PPP3/calcium-regulated phosphatase, which maintains TFEB in a phosphorylated inactive state [144].

Recent evidence has demonstrated a substantial association between lysosomes and ferroptosis. Lysosomal inhibitors, such as ammonium chloride, pepsin inhibitor A, and bafilomycin A can prevent ferroptosis induced by Erastin or RSL3 [17]. This implies that the STAT3/CTSB axis serves as a pivotal mediator between autophagy and ferroptosis. Erastin activates the STAT3/CTSB axis to induce lysosomal membrane permeabilization, which leads to leakage of CTSB mediating the lysosome-nucleus communication pathway and thus its accumulation in the nucleus, resulting in DNA damage and histone H3 cleavage, and the activation of the interferon-STING1-dependent DNA sensor pathway, prompting the STING1-containing endoplasmic reticulum-Golgi intermediate region compartment to act as an autophagosome membrane source and induce autophagy-dependent ferroptosis [17,[145], [146], [147]]. The pharmacological tissue protease inhibitor CA-074Me suppresses ferroptosis by blocking CTSB [148]. However, CTSB also prevents ferroptosis by catabolizing albumin through a lysosome-dependent pathway, which boosts cysteine levels, promoting glutathione synthesis and reducing lipid peroxidation [149]. Additionally, sorafenib is thought to promote the dephosphorylation of STAT3 through Src homologous region 2 structural domain-containing phosphatase-1, decreasing Myeloid leukemia sequence 1 (MCL-1) expression and thereby weakening the association between MCL-1 and Beclin-1, ultimately inducing Beclin-1/SLC7A11-dependent ferroptosis [124]. STAT3 has been demonstrated to inhibit ACSL4, thereby restricting the esterification of PUFAs to acyl-coenzyme A and preventing lipid peroxidation, which inhibits ferroptosis [150]. Activation of STAT3 by integrin subunits alpha (ITGA) 6 and ITGB4 promotes cell survival during Erastin treatment [27]. This reveals that multiple pathways converge on a complex signaling network, with STAT3 serving as a central hub to regulate autophagy and ferroptosis.

### 3.4 HMGB1

HMGB1, a non-histone nuclear protein, operates as a DNA chaperone, preserving chromosome structure and function while regulating gene transcription, replication, and recombination [151]. HMGB1, the second most abundant nuclear protein, is capable of translocating from the nucleus to the cytoplasm or extracellular space under stress. In this manner, it functions as a damage-associated molecular pattern molecule (DAMP) to mediate inflammatory and immunological responses [152]. Previous studies have indicated that HMGB1 may activate Nrf2 and STAT3 pathways while regulating CTSB release [153,154]. CTSB nuclear translocation damages DNA and releases HMGB1, which contributes to ferroptosis events [155]. This reveals that HMGB1 is crucial in regulating autophagy and ferroptosis.

HMGB1 triggers autophagy through various pathways depending on its subcellular localization [156]. Nuclear HMGB1 enhances the expression of HSP family B (small) member 1 (HSPB1), regulates actin filament assembly in autophagic membrane dynamics, and promotes cytoskeletal reorganization. Cytoplasmic HMGB1 could stimulate the formation of autophagosome and enhance the autophagic flux through an intramolecular disulfide bond (C23/45) binding to Belcin-1. Extracellular HMGB1 further promotes autophagy by activating the PIK3C3 complex or inducing the expression of HSP27 [25,157]. Additionally, HMGB1 activates the downstream the rat sarcoma (RAS)-JNK/p38 pathway by MAPK, leading to increased transferrin receptor (TFRC) expression and elevated intracellular serum transferrin or lactoferrin levels, ultimately causing iron overload and triggering ferroptosis [157]. Autophagy and ferroptosis interact closely through HMGB1, which can induce ferroptosis by promoting ferritin autophagy [17]. It has been demonstrated that the release of HMGB1 can be triggered by various ferroptosis activators, while the inhibition of autophagy can effectively block this process. This can be performed through the silencing of ATG family proteins, such as ATG5, ATG7, and ATG12, or by administering lysosomal inhibitors like chloroquine and bafilomycin A1 [151,158,159]. Conversely, autophagy could enhance the release of HMGB1 during ferroptosis by reducing the acetylation of HMGB1 by histone deacetylase [151]. Modulating HMGB1 and ferroptosis holds significant translational potential. Glycyrrhizin (GL), an HMGB1 inhibitor, mitigates neuronal ferroptosis and reduces mitochondrial damage, contributing to reducing neuroinflammation in neonatal hypoxic-ischemic brain damage (HIBD) in rats. Thus, it suggests novel HIBD prevention and treatment strategies [160].

## 4 Autophagy and ferroptosis interaction in CVDs and potential treatments

**Fig. 10:**
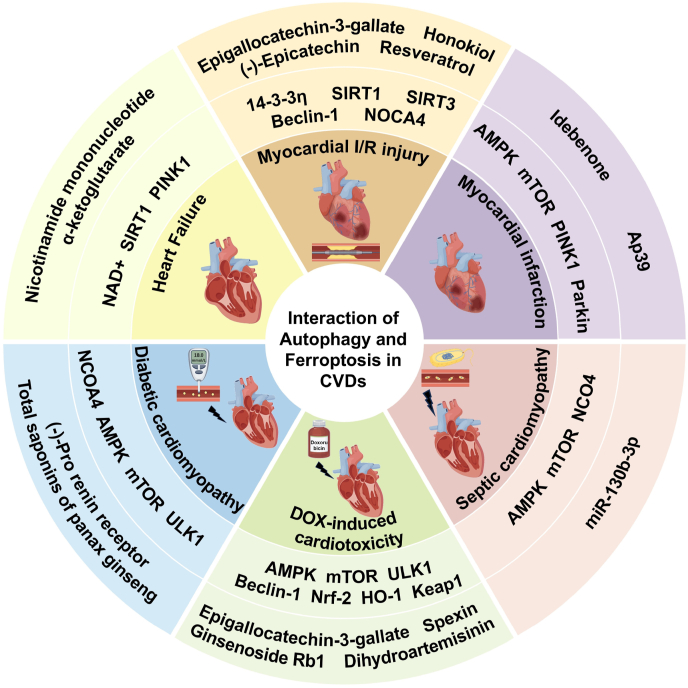
Interaction of ferroptosis and autophagy in CVDs and potential therapeutic drugs.

Recently, numerous studies have indicated a potential interaction between autophagy and ferroptosis in CVDs. Therapeutic strategies employing autophagy and ferroptosis as common targets have also garnered significant interest. Herein, we review and summarize the interaction between autophagy and ferroptosis and related therapeutic strategies in various cardiovascular diseases (Table 1) (Fig. 10).

**Table 1:** Interaction between autophagy and ferroptosis in CVDs and potential regulatory treatments in *in vitro* and preclinical models.

Disease	Treatments	Dose	Molecules /pathway	Function	Therapeutic effect	Ref.
MI	Idebenone	*In vitro:* 0.1, 0.2, 0.5, 1, 2 μmol/L (H9c2 cell) *In vivo:* 100 mg/kg/d for 3 days or 4 weeks	AMPK /mTOR	Inhibition of autophagy-dependent ferroptosis	Alleviating myocardial infarction and maintaining cardiac function	[166]
MI	Ap39	*In vitro:* 100 nM for 48h (H9c2 cell) *In vivo:*	PINK1 /Parkin	Inhibition of excessive mitophagy-mediated ferroptosis	Improving MI-induced cardiac fibrosis	[163]
MI	Overexpression of microRNA 30d (miR-30d)	–	ATG5	Inhibition of ferritinophagy-mediated ferroptosis	Improving cardiomyocyte death after MI	[167]
MI/RI	(−)-Epicatechin (EPI)	*In vitro*: 2, 5, 10 μM for 24 h (H9c2 cell) *In vivo*: 1–2 mg/kg/d for 15 days	USP14 /Beclin-1 /NCOA4	Inhibition of autophagy-dependent ferroptosis	Improving I/R-induced abnormal electrocardiogram waveform and reducing infarct size	[174]
MI/RI	Si-ELAVL1	–	FOXC1 /ELAVL1 /Beclin-1	Inhibition of autophagy-dependent ferroptosis	Improving lipid peroxidation and reducing infarct size	[175]
MI/RI	Epigallocatechin-3-gallate (EGCG)	*In vitro*: 10 μM for 48 h (H9c2 cell) *In vivo*: 20 mg/kg/d for 6 weeks	14-3-3η	Attenuating ferroptosis, apoptosis, and autophagy	Reducing ROS levels, decreasing infarct size, and maintaining cardiac function	[176]
MI/RI	Resveratrol (Res) Honokiol (HKL)	*In vitro*: 20 nmol for 24 h (H9c2 cell)	SIRT1 /SIRT3 /PINK1 /Parkin	Reversal of mitophagy imbalance and inhibition of ferroptosis	–	[177]
HF	Nicotinamide mononucleotide (NMN)	*In vitro:* 1 mM for 48 h *In vivo:* 5 mg /d	NAD+	Restoring lysosomal function, enhancing autophagy and inhibiting lysosomal dysfunction-mediated ferroptosis	Improving heart failure	[182]
HF	α-ketoglutarate (AKG)	*In vitro:* 2 μM for 24h (NRCMs) *In vivo:* 2 % AKG was added to the drinking water	NAD+ /SIRT1	Promotion of cardiomyocyte autophagy and inhibition of ferroptosis	Mitigating myocardial hypertrophy, fibrosis and cardiac insufficiency	[183]
DIC	Epigallocatechin-3-gallate (EGCG)	*In vitro*: 20 μM for 24 h (H9c2 cell and NRCMs) *In vivo*: 20 mg/kg/d for 6 weeks	AMPKα2 /mTOR /ULK1	Attenuating ferroptosis and apoptosis through activating adaptive autophagy	Maintaining mitochondrial function, improving DOX-induced cardiac dysfunction and fibrosis	[191]
DIC	SPATA2 siRNA	*In vitro*: 100 pmol for 24 h (H9c2 cell)	SPATA2 /CYLD /USP14 /NCOA4	Inhibition of ferroptosis via ferritinophagy	Knockdown of SPATA2 could reduce ferritinophagy and ferroptosis	[192]
DIC	Spexin (SPX)	*In vitro*: 1 μM for 12 h (NRCMs) *In vivo*: 50 μg/kg/d for 4 weeks	Beclin-1	Inhibition of excessive autophagy-induced ferroptosis	Improving cardiac function, reducing biomarkers of myocardial injury, and inhibiting cardiac fibrosis	[193]
DIC	Ginsenoside Rb1 (Rb1)	*In vivo*: 40 mg/kg/d for 7 days	AMPK /mTOR /Nrf2	Suppression of autophagy and ferroptosis	Attenuating DOX-induced cardiac dysfunction, myocardial hypertrophy and interstitial fibrosis	[194]
DIC	Dihydroartemisinin (DHA)	*In vitro*: 5, 10, 20 μM for 24 h (H9c2 cell) *In vivo*: 5, 25, 50 mg/kg/d for 5 weeks	Nrf2 /HO-1	Alleviating ferroptosis by promoting autophagosome degradation	Improving cardiac function, reducing biomarkers of myocardial injury, and alleviating inflammatory infiltration	[195]
DCM	Knockdown of PRR	–	NCOA4	Inhibition of ferritinophagy-mediated ferroptosis	Improving cardiac function	[198]
DCM	Total saponins of panax ginseng (TSPG)	*In vivo:* 10 or 30 mg/kg/d for 8 weeks	AMPK /mTOR /ULK1	Inhibition of ferritinophagy-mediated ferroptosis	Improving diabetes-induced myocardial injury	[199]
SCM	–	–	NCOA4	Inducing ferritinophagy-mediated ferroptosis	Induction of septic cardiomyopathy	[202]
SCM	Overexpression of miR-130b-3p	–	ACSL4 /AMPK /mTOR	Inhibition of autophagy-dependent ferroptosis	Inhibiting collagen deposition, improving myocardial inflammation and cardiac function	[204]
HFD-related cardiac remodeling	Piperlongumine (PLG)	*In vitro:* 5 μM or 7.5 μM for 24 h (H9c2 and AC16 cell) *In vivo:* 5 mg/kg/d for 12 weeks	STAT3	Suppression of ferritinophagy-mediated ferroptosis	Attenuating ventricular remodeling and improving cardiac function	[32]
Pm2.5-related cardiac fibrosis	–	–	YY1 /NCOA4	Inducing ferritinophagy-mediated ferroptosis	Induction of cardiac fibrosis	[208]
PM2.5-related cardiac hypertrophy	MitoQ	*In vitro:* 0.5 μM for 2h (AC16 cell) *In vivo:* 5 mg/kg for 4 weeks	DHODH /NCOA4	Inhibition of ferritinophagy and mitophagy crosstalk-mediated ferroptosis	Mitigating PM2.5-triggered cardiac dysfunction and hypertrophy	[207]

**Abbreviations:** MI, myocardial infarction; MI/RI, myocardial ischemia-reperfusion injury; HF, heart failure; DIC, doxorubicin-induced cardiotoxicity; DCM, diabetic cardiomyopathy; SCM, septic cardiomyopathy; HFD, high-fat diet; NRCMs, Neonatal rat cardiomyocytes; AMPK, AMP-activated protein kinase; mTOR, mammalian target of rapamycin; PINK1, PTEN induced putative kinase 1; USP, ubiquity specific peptidase; NCOA4, nuclear receptor coactivator 4; ROS, reactive oxygen species; ELAVL1, embryonic lethal-abnormal vision like protein 1; FOXC1, forkhead box C1; SIRT, sirtuin; NAD+, nicotinamide adenine dinucleotide; ULK1, unc-51-like kinase 1; SPATA2, spermatogenesis-associated protein 2; CYLD, cylindromatosis; Nrf2, nuclear factor erythroid 2-related factor 2; HO-1, heme oxygenase-1; PRR, pro renin receptor; ACSL4, acyl-CoA synthetase long-chain family member 4; STAT3, signal transducer and activator of transcription 3; YY1,Yin Yang 1; DHODH, dihydroorotate dehydrogenase.

### 4.1 Myocardial infarction (MI)

As the leading cause of cardiac sudden death, MI leads to cardiomyocyte death and causes subsequent cardiac remodeling and heart failure. Multiple cell death pathways involve in the pathophysiology of MI, including mitochondria-dependent necrosis, ferroptosis, autophagy and pyroptosis [161]. Myocardial ischemia and hypoxia lead to excess ROS generation, and ROS-mediated lipid peroxidation acts as a signal that initiates multiple forms of cell death, including ferroptosis and autophagy. The role of autophagy in MI still remains controversial. It has been found that enhanced autophagy could ameliorate post-MI cardiac remodeling, remove damaged mitochondria, and maintain the level of ATP. Nevertheless, excessive activated autophagy triggers a series of adverse intracellular effects, including complete intracellular mitochondrial clearance, inadequate ATP synthesis, impaired clearance of harmful metabolites, and elevated ROS levels, thereby affecting cardiac systolic function [162,163]. Meanwhile, ferroptosis represents a significant contributor to myocardial injury following MI. Studies have shown that ferroptosis inducers such as ferric iron could significantly induce the cardiomyocyte death, while ferrostatin-1 and other ferroptosis inhibitors can significantly reduce MI [164,165]. The complex regulatory mechanism of ferroptosis in MI needs to be further studied. Autophagy potentially exerting a pivotal role in MI-induced ferroptosis of cardiomyocytes. AMPK and mTOR were identified as key molecules in the idebenone-mediated inhibition of autophagy and ferroptosis. Experimental evidence has demonstrated that idebenone could inhibit excessive autophagy via the ROS-AMPK-mTOR pathway, thus attenuating ferroptosis and ameliorating post-MI cardiac function [166]. Moreover, PTEN induced putative kinase 1 (PINK1)/Parkin-mediated mitophagy was proven to be activated during MI, and mitochondria-targeted hydrogen sulphide (H_2_S) donor AP39 inhibited excessive mitophagy, maintained mitochondrial membrane potential, reduced ROS generation, prevented cardiomyocyte ferroptosis and ameliorated cardiac fibrosis in MI rats [163]. Tang et al. demonstrated that autophagy after MI may promote cardiomyocyte ferroptosis by degrading ferritin and increasing the dissociative iron content in cardiomyocytes. ATG5 is a downstream target gene of microRNA-30d (miR-30d). Upon overexpression of miR-30d, miR-30d binds to ATG5 to inhibit autophagy and reduce cardiomyocyte ferroptosis [167]. Collectively, these findings provide evidence for the interaction between autophagy and ferroptosis in pathological process of MI.

### 4.2 Myocardial ischemia-reperfusion injury (MI/RI)

In-time reperfusion strategies following MI, including percutaneous coronary intervention (PCI) and endovascular thrombolysis, have been demonstrated to effectively reduce the mortality and improve prognosis in patients with MI. However, although reperfusion therapy improves myocardial blood supply and preserves myocardium in the ischemic border zone, it also induces myocardial ischemia-reperfusion injury [168]. It is established that several potential mechanisms are involved in the pathophysiology of MI/RI, including Ca^2+^ overload, excessive ROS production, and microthrombus formation [169]. Various cell death pathways are involved in MI/RI, including apoptosis, necroptosis, autophagy, necrosis, pyroptosis, and ferroptosis [170]. Recent preclinical research has demonstrated that ferroptosis inhibitors Liproxstatin-1 and Ferrostatin-1 could decrease the levels of voltage-dependent anion channel (VDAC1) or activate Nrf2, respectively, thus upregulating the levels of GPX4 and protecting against myocardial MI/RI [171,172]. Notably, increasing experimental evidence have suggested that the imbalance of autophagy and the onset of ferroptosis play a pivotal role in MI/RI, and the interaction of autophagy and ferroptosis might represent a novel and essential target for the protection against MI/RI [[173], [174], [175], [176], [177]].

Currently, USP, Beclin-1, and NCOA4 are considered to be the key molecules protecting against MI/RI through autophagy and ferroptosis interaction. Previous studies have shown that resveratrol (Res) and (−)-Epicatechin (EPI) could inhibit myocardial excessive autophagy and ferroptosis through the USP14/19-Beclin-1-NCOA4 pathway, thus attenuating oxidative stress and improving MI/RI [173,174]. Moreover, the knockdown of the upstream gene ELAVL1 resulted in a reduction in Beclin-1 expression, and inhibited autophagy-dependent ferroptosis, thereby ameliorating lipid peroxidation and reducing infarct size in the MI/RI model [173,175]. Myocardial 14-3-3η protein has been identified as a key regulator of autophagy and mitochondria-mediated apoptosis in MI/RI [178,179]. Recently, Hu et al. demonstrated that epigallocatechin-3-gallate (EGCG) pretreatment could upregulate the expression of 14-3-3η. Subsequently, the ratio of LC3-II/I was downregulated, mitochondrial ROS production was reduced and the expression of Bcl2 and Bcl-2-associated X protein (Bax) was downregulated, ultimately inhibiting MI/RI-induced myocardial autophagy, apoptosis, and ferroptosis. This finding indicated that 14-3-3η might serve as a common molecular mediator of apoptosis, ferroptosis and autophagy in MI/RI [176]. Additionally, the interaction between mitophagy and ferroptosis have been further investigated in MI/RI. Sirtuin1 (SIRT1) and Sirtuin3 (SIRT3) are responsible for mitochondrial quality control, particularly for maintaining the mitochondrial homeostasis. Liao et al. demonstrated that MI/RI resulted in the silencing of the SIRT1/SIRT3/PINK1/Parkin axis and the suppression of mitophagy. Dysfunctional mitophagy caused an accumulation of ROS in mitochondria, which in turn exacerbated ferroptosis in cardiomyocytes [177]. Furthermore, the administration of resveratrol and honokiol was proven to reverse these alterations, suggesting novel avenues for the regulation of mitophagy and ferroptosis in MI/RI.

### 4.3 Heart failure (HF)

HF is a clinical syndrome of cardiac dysfunction resulting from various structural or functional cardiac disorders, which is often the end stage of multiple cardiac diseases. The pathogenesis of HF is multifaceted, involving a variety of cellular and molecular mechanisms, including apoptosis, necrosis, and autophagy in cardiomyocytes [180]. Autophagy and ferroptosis could be observed in the HF rat model. Autophagy exerts a multifaceted effect in HF. In the initial phases of HF, oxidative stress and inflammation could trigger cardiomyocyte autophagy. Moderate autophagy removes damaged organelles, downregulates inflammatory factors and ROS, thereby reducing cardiomyocyte apoptosis and preventing deterioration of cardiac function. While in the advanced stages of HF, the accumulation of conformationally abnormal proteins, dysfunctional organelles and ROS in cardiomyocytes triggers excessive autophagic responses. Although overactivated autophagy serves to eliminate harmful factors, it also results in the damage of organelles and proteins required to maintain normal cellular function. In addition, dysfunctional autophagy may also occur in the late HF phase, possibly due to cellular metabolic depletion that impairs autophagic pathway function, which impedes the clearance of harmful factors. These leads to the promotion of cardiomyocyte injury and apoptosis, ultimately accelerating cardiac adverse remodeling and deterioration of cardiac function [180]. In addition, recent studies have indicated that ferroptosis may be involved in cardiomyocyte damage, which ultimately leads to HF [181]. Normal functioning lysosomes are involved in the process of autophagy. It has been demonstrated that mitochondrial dysfunction-induced lysosomal damage could precipitate iron accumulation and induce ferroptosis. Nicotinamide mononucleotide (NMN) could increase nicotinamide adenine dinucleotide (NAD+) levels, improve lysosomal function and reduce damaged lysosomes, thereby improving autophagy, as well as inhibiting lysosomal dysfunction-mediated ferroptosis and improving HF [182]. Yu et al. found that α-ketoglutarate (AKG) was capable of enhancing intracellular NAD + levels, upregulating the expression of SIRT1 and downstream proteins PINK1 and phospholipid hydroperoxidase GPX4, thereby promoting mitophagy in cardiomyocytes, inhibiting ferroptosis, and attenuating myocardial hypertrophy, fibrosis and cardiac dysfunction [183]. Moreover, Chen et al. demonstrated that the toll-like receptor 4 (TLR4)- NADPH oxidase (NOX)4 pathway is closely associated with ferroptosis and autophagy in HF. Regulation of TLR4-NOX4 pathway can simultaneously inhibit ferroptosis and autophagy, thus ameliorating cardiac dysfunction, but the specific crosstalk remains to be further investigated [184].

### 4.4 Doxorubicin-induced cardiotoxicity (DIC)

Doxorubicin (DOX) is a broad-spectrum chemotherapeutic agent commonly employed in the management of lymphoma and breast cancer. Nevertheless, DOX-induced cardiotoxicity is observed in approximately one-quarter of patients in clinical practice, which constrains its clinical application [185]. The current researches indicate that the pathophysiological mechanisms of DIC are primarily associated with autophagy, apoptosis, ferroptosis, and pyroptosis, which occur concurrently and interact with each other, significantly complicating the treatment of DIC [[186], [187], [188]]. Recent advances have demonstrated that DOX induces iron overload in cardiomyocytes by participating in iron uptake, storage, and promoting iron accumulation in mitochondria [189]. On the other hand, DOX has been observed to significantly downregulate GPX4, resulting in GSH/GPX4 dysregulation in cardiomyocytes, which might play a pivotal role in the pathogenesis of DIC [190]. Additionally, the presence of ROS and lipid peroxides, such as 4-hydroxynonenal and malondialdehyde, accompanied by ferroptosis, was significantly increased in cardiomyocytes cultured with DOX [190]. Consequently, these results suggest that DOX may trigger ferroptosis, which in turn may lead to cardiotoxicity. In contrast, the role of autophagy in DIC is controversial, as the results of studies utilizing pharmacological or genetic approaches to inhibit autophagy have been inconsistent in DIC. Some studies reported protective effects, whereas others have shown exacerbation of DIC. Recent evidence suggests that DOX initially induces autophagy, which scavenges damaged mitochondria and ROS. However, subsequent blockade of autophagy by DOX leads to the accumulation of undegraded autolysosomes, which in turn leads to ROS generation and exacerbation of DIC [186]. Consequently, the extent of autophagy impacts the level of ROS during the pathophysiologic process of DIC. Since the level of ROS has been demonstrated to affect the elicitation of lipid peroxidation and ferroptosis, the dual regulatory effect of autophagy on ROS might exacerbate or alleviate ferroptosis in DIC.

He et al. demonstrated that EGCG pretreatment upregulated AMPKα2, activated adaptive autophagy, and maintained mitochondrial function, thereby protecting cardiomyocytes from DOX-induced ferroptosis and apoptosis [191]. This provided experimental evidence that enhanced autophagy could antagonize ferroptosis in DIC. Conversely, knockdown of spermatogenesis-associated protein 2 (SPATA2) inhibited ferritinophagy through the SPATA2/USP/NCOA4 pathway, thus ameliorating DOX-induced ferroptosis [192]. As co-regulatory molecules of autophagy and ferroptosis, Beclin-1 and Nrf2 serve as essential targets for the treatment of DIC. Spexin has been demonstrated to inhibit excessive autophagy-induced ferroptosis by upregulating Beclin-1 [193]. Zhai et al. have demonstrated that Ginsenoside Rb1 could activate Nrf2, upregulate the levels of FTH1 and GPX4, and simultaneously inhibit cardiomyocyte autophagy and ferroptosis, thereby improving DOX-induced cardiac dysfunction and fibrosis [194]. Furthermore, dihydroartemisinin was found to activate Nrf2 and HO-1, facilitate autophagosome clearance, attenuate lysosomal destruction, and alleviate DOX-induced ferroptosis [195]. In a recent study, Yin et al. demonstrated that astaxanthin (ASX) possesses the capacity to downregulate Beclin-1 and upregulate SLC7A11 and GPX4, thereby inhibiting autophagy and ferroptosis and ameliorating DIC [196]. The existing experimental evidence revealed the dual roles of the antagonistic and synergistic effects of autophagy and ferroptosis in DIC, offering potential clinical strategies for prevention and treatment of DIC.

### 4.5 Diabetic cardiomyopathy (DCM)

DCM is a common complication of diabetes, characterized by cardiac structural abnormalities and dysfunction, including myocardial fibrosis, ventricular dilatation and cardiac dysfunction. DCM is acknowledged as a principal contributor to heart failure and mortality among patients with diabetes. Cellular death pathways in DCM have been confirmed to encompass apoptosis, necrosis, autophagy and ferroptosis [197]. Among them, the interaction between autophagy and ferroptosis plays an important role in the development of DCM. As a multifunctional receptor that binds to renin or reninogen, pro renin receptor (PRR) is responsible for maintaining the stability of autophagosomes and facilitating the fusion of autophagosomes with lysosomes. Moreover, it is established that PRR is intimately associated with myocardial fibrosis, inflammation, and deterioration of cardiac function in DCM. The knockdown of PRR has been demonstrated to inhibit NCOA4-mediated ferritinophagy, reduce ROS production and lipid peroxidation, and ultimately attenuate the ferroptosis of cardiomyocytes in DCM, thus improving cardiac function. Furthermore, Zhang et al. demonstrated that ferroptosis inhibitor Fer-1 inhibits high glucose-induced ferroptosis can improve DCM in mice. Zhang et al. demonstrated that ferroptosis inhibitor ferrostatin-1 could inhibit high glucose-induced ferroptosis can improve DCM in mice [198]. Additionally, total saponins of panax ginseng (TSPG) has been identified as a potential therapeutic agent for DCM, which could improve diabetes-induced myocardial damage by regulating AMPK/mTOR/ULK1-mediated ferritinophagy [199]. The aforementioned experimental evidence suggests that autophagy-mediated ferroptosis might represent a novel target for the treatment of DCM.

### 4.6 Septic cardiomyopathy (SCM)

SCM is a sepsis-related reversible myocardial dysfunction, with a high mortality rate and an unfavorable prognosis. SCM appears to be associated with several forms of cell death including autophagy, apoptosis, and ferroptosis [200,201]. During the sepsis, uncontrolled autophagy occurs due to acute stress [200]. Recent findings also suggest that ferroptosis might be involved in lipopolysaccharide (LPS)-induced cardiac injury and dysfunction [202]. Moreover, it has been demonstrated that LPS instigates the initiation of autophagy in cardiomyocytes by activating the Beclin-1/ATG5/ATG7/calcineurin signaling pathway, promotes the generation of ROS, and affects mitochondrial homeostasis, ultimately leading to ferroptosis. Catalase overexpression could attenuate autophagy and ferroptosis, effectively rescuing LPS-mediated oxidative stress, apoptosis, autophagy, ferroptosis and mitochondrial damage [203]. Furthermore, ferritinophagy-triggered ferroptosis plays an important role in sepsis-induced cardiac injury. NCOA4 could directly interact with ferritin to degrade it in a ferritin-dependent manner, resulting in the release of large amounts of dissociative iron, inducing ferroptosis in cardiomyocytes and SCM [202]. Another study showed that miR-130b-3p could inhibit ferroptosis through direct and indirect pathway. On the one hand, it can be directly targeted to inhibit activity of ACSL4. On the other hand, it alleviated ferroptosis by inhibiting cardiomyocyte autophagy through suppression of the AMPK/mTOR signaling pathway. Consequently, the overexpression of miR-130b-3p inhibited autophagy-dependent ferroptosis, suppressed collagen deposition, ameliorated myocardial inflammation, and improved cardiac function [204].

### 4.7 Others

In recent years, there has been considerable concern about the dramatic increase in obesity and its association with cardiac abnormalities. High fat diet (HFD) intake is among other reasons for obesity and cardiac abnormalities, causes obesity and cardiac abnormalities which typically include cardiac hypertrophy, diminished ventricular compliance and cardiac dysfunction, accompanied by obvious mitochondrial damage and various forms of cell death in cardiomyocytes [81] Previous study revealed that up-regulation of STAT3 phosphorylation in mice receiving HFD promotes NCOA4-mediated ferritinophagy, leading to iron overload and increased ROS levels. This ultimately triggers ferroptosis in cardiomyocytes. As an inhibitor of STAT3, piperlongumine (PLG) could impede STAT3-mediated ferritinophagy and ferroptosis, reducing HFD-induced cardiotoxicity and improving ventricular remodeling and cardiac function [32].

Air pollution is regarded as one of the major environmental health risks [205]. Epidemiological study has demonstrated the association between chronic exposure to Fine particulate matter (PM2.5) and the significantly increased risk of developing CVDs [206]. Myocardial hypertrophy and fibrosis are the major causes of CVDs induced by PM2.5 [207]. It was established that following exposure to PM2.5, iron regulatory molecules (NCOA4, FTH1) were abnormally expressed, indicating the activation of ferritinophagy and imbalance of iron homeostasis. Concurrently, mitophagy was initiated and accompanied by mitochondrial dysfunction. The activation of ferritinophagy preceded mitophagy, as evidenced by the time series of PM2.5 exposure. MitoQ, a mitochondrial-targeted antioxidant, has been proven to inhibit ferroptosis via PM2.5-triggered ferritinophagy, mitophagy crosstalk-mediated iron homeostasis imbalance and alleviate PM2.5-induced cardiac dysfunction and myocardial hypertrophy [207]. Moreover, Hu et al. demonstrated that transcription factor YY1 may regulate ferritinophagy-dependent ferroptosis and promote cardiac fibrosis by regulating NCOA4 protein interactions after PM2.5 exposure [208]. The aforementioned studies illustrate the significant function of the crosstalk between autophagy and ferroptosis in the context of CVDs, and provide preliminary evidence that targeting this crosstalk might offer promising prospects for the treatment of CVDs.

Since ROS play a pivotal role in the crosstalk of autophagy and ferroptosis, numerous antioxidants could modulate this pathophysiologic process and improve the outcome of CVDs. Current preclinical studies have confirmed the therapeutic effects of several antioxidants on CVDs, mainly including idebenone, EGCG, resveratrol, honokiol, NMN, ginsenoside Rb1, dihydroartemisinin, and MitoQ. It is noteworthy that some of these antioxidants have been subjected to relevant clinical trials or pilot studies confirming their beneficial effects on CVDs. In a randomized controlled trial, K. Magyar et al. demonstrated that resveratrol (10 mg daily for three months) could improve left ventricular diastolic and endothelial function in patients with myocardial infarction, and have a tendency to improve left ventricular ejection fraction [209]. In 2022, Masoumi-Ardakani et al. found that MitoQ improved cardiac function, oxidative stress, and inflammation levels in a cohort of 52 patients with hypertension [210]. Furthermore, evidence suggests that EGCG is capable of lowering blood pressure, improving heart rate variability, and reducing left ventricular myocardial mass in patients suffering from heart failure (myocardial amyloidosis), thus exerting cardioprotective effects [211,212]. Nevertheless, the cardioprotective effects of these drugs were only preliminarily demonstrated in these clinical studies, and further investigation is required to elucidate the specific mechanisms of autophagy and ferroptosis.

## 5 Conclusion and perspectives

In the pathophysiological process of CVDs, autophagy has been observed to exert a dual effect, either cytoprotective or lethal, depending on the degree of activation. However, either cytoprotective or lethal autophagy commonly predominates at a given stage of disease development. In recent years, the interaction and crosstalk between autophagy and ferroptosis have received extensive attention in CVDs. Autophagy could protect cells from ferroptosis or conversely induce autophagy-dependent ferroptosis. The current co-regulatory molecules of these two pathophysiological processes are primarily including Beclin-1, Nrf2, STAT3, and HMGB1. Preliminary studies have demonstrated that therapeutic strategies targeting both autophagy and ferroptosis might enhance the prognosis of CVDs and offer promising avenues for novel therapeutic approaches. However, the extant evidence also indicated that ferroptosis could occur independently of cellular autophagy, and the relationship between these two remains to be investigated. Further researches are required to elucidate the relationship and interaction between autophagy and ferroptosis and identify appropriate regulatory mechanisms to achieve a balance between autophagy and ferroptosis, thereby protecting cardiomyocytes and optimizing the prognosis of CVDs. Moreover, current therapeutic drugs primarily exert their cardioprotective effects by influencing ferritinophagy and mitophagy, and there is currently a lack of research on the interaction between other subtypes of autophagy and ferroptosis in the treatment of CVDs. Further researches should be directed at elucidating the role of the interaction between other subtypes of autophagy and ferroptosis in CVDs and the development of related drugs. Furthermore, current studies on the interaction of ferroptosis and autophagy for the treatment of CVDs are dominated by non-specific drugs. These drugs have the capacity to influence the contractual targets and interacting processes associated with autophagy and ferroptosis. However, there is a paucity of specific drugs against these co-regulators of autophagy and ferroptosis for the treatment of CVD. Consequently, developing targeted pharmacological agents could represent a significant future research direction, with considerable potential for clinical translation.

## CRediT authorship contribution statement

**Changhao Hu:** Writing – review & editing, Writing – original draft, Visualization, Investigation. **Siying Gao:** Writing – review & editing, Writing – original draft, Visualization, Investigation. **Xinyi Li:** Writing – review & editing, Writing – original draft, Investigation, Conceptualization. **Kaiqing Yang:** Investigation. **Ye Cheng:** Investigation. **Wei Guo:** Investigation. **Huijun Wu:** Investigation. **Xueqin Cheng:** Investigation. **Weiwen Zhao:** Investigation. **Yuxuan Kong:** Investigation. **Haoyuan Hu:** Writing – review & editing, Writing – original draft, Investigation, Conceptualization. **Songyun Wang:** Writing – review & editing, Conceptualization.

## Funding

This work was funded by the National Natural Science Foundation of Hubei Province (JCZRYB202400863), the Interdisciplinary Innovative Talents Foundation from Renmin Hospital of Wuhan University (NO. JCRCYG-2022-001), the Fundamental Research Funds for the Central Universities (NO.2042023kf0182), and the Open research fund of State Key Laboratory of Cardiovascular Diseases (No.08) .

## Declaration of competing interest

The authors declare that they have no known competing financial interests or personal relationships that could have appeared to influence the work reported in this paper.

## Data Availability

No data was used for the research described in the article.
